# A set of indicators for decomposing the secular increase of life expectancy

**DOI:** 10.1186/1478-7954-8-18

**Published:** 2010-06-09

**Authors:** Valentin Rousson, Fred Paccaud

**Affiliations:** 1 Institute for Social and Preventive Medicine (IUMSP), University Hospital and Faculty of Medicine, Lausanne, Switzerland

## Abstract

**Background:**

The ongoing increase in life expectancy in developed countries is associated with changes in the shape of the survival curve. These changes can be characterized by two main, distinct components: (i) the decline in premature mortality, i.e., the concentration of deaths around some high value of the mean age at death, also termed rectangularization of the survival curve; and (ii) the increase of this mean age at death, i.e., longevity, which directly reflects the reduction of mortality at advanced ages. Several recent observations suggest that both mechanisms are simultaneously taking place.

**Methods:**

We propose a set of indicators aiming to quantify, disentangle, and compare the respective contribution of rectangularization and longevity increase to the secular increase of life expectancy. These indicators, based on a nonparametric approach, are easy to implement.

**Results:**

We illustrate the method with the evolution of the Swiss mortality data between 1876 and 2006. Using our approach, we are able to say that the increase in longevity and rectangularization explain each about 50% of the secular increase of life expectancy.

**Conclusion:**

Our method may provide a useful tool to assess whether the contribution of rectangularization to the secular increase of life expectancy will remain around 50% or whether it will be increasing in the next few years, and thus whether concentration of mortality will eventually take place against some ultimate biological limit.

## Background

Life expectancy almost doubled in developed countries during the 20th century. This increase was initially related to the reduction in infant mortality. After the 1950 s, the increase was related to the decline in mortality in old age [1]. In both phases, the evolution is usually related to the combination of a massive improvement in the social and physical environment and to an improvement in health care. Whether the former improvement had more effect on the decline of mortality than the latter (as suggested by McKeown's theory) is still a matter of debate [2]. No strong, comprehensive theory is yet available to fully understand the past trends, and hence, the current and future evolution of human longevity. However, several paradigms have been proposed to capture the underlying mechanisms at stake. One of them was provided by Fries in 1980 [3], relating the secular increase of life expectancy to the postponement of age of death. This corresponds to the strong decline (or even the fading) of premature mortality and morbidity, and to the concentration (or compression) of deaths around some fixed, optimal mean age at death value, estimated at 85 years. This evolution was coined as "rectangularization," suggesting that the final stage of the survival curve will be a perfect rectangle.

This paradigm developed by Fries was mainly based on visual inspection. Several authors developed quantitative indicators to capture the shape of the survival curves [4-8]. Most confirmed a concentration of age at death around high mean values with a decreasing variability. However, some papers showed a slowdown of rectangularization during the last decades, sometimes with a decline resuming after a plateau [9], while others found an increase in the variability of age at death over 60 years [10,11]. In the same line, others showed a continuous increase of the maximal age at death in the last century [1,12], with no signs of a finite life-span limit [13]. In the latter paper, Wilmoth explicitly suggested that rectangularization of the survival curve and increase of longevity are in fact occurring simultaneously, i.e., that the distribution of age at death could become more and more compressed while also shifting further and further to the right. In order to evaluate and monitor this hypothesis, we propose in this paper a set of quantitative, nonparametric indicators, namely a rectangularity index and a longevity index, to quantify and disentangle the respective contributions of rectangularization and longevity increase to the secular increase of life expectancy.

## Methods

Wilmoth and Horiuchi [5] reviewed 10 measures characterizing either the rectangularity of a survival curve or the variability of age at death. One of these was coined "moving rectangle" and constructed as follows. Let S*(*t*) be the survival curve from a cohort of interest, and let  be a very high quantile of the distribution of age at death, such that  (Wilmoth and Horiuchi considered *Q *= .999). Consider a rectangle with lower-left and upper-right corner coordinates (0,1 - *Q*) and (, 1), thus of dimension *Q*. A straightforward measure of rectangularity is given by the proportion of this rectangle lying below the survival curve, defined as:

Our approach is based on this indicator, and is developed in three steps as follows.

**Figure 1:**
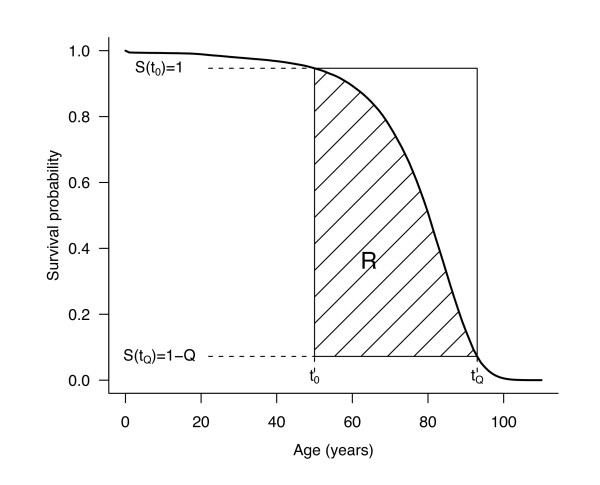
**Example of survival curve with rectangularity index *R* and longevity index *t_Q_***. These indexes are calculated from the conditional survival curve *S*(*t*) for those people reaching the age of *t*_0_, such that *S*(*t*_0_ ) = 1 and *S*(*t_Q_*) = 1 - *Q*. On that example, *t*_0_ = 50, *Q* = 0.9, *R* = 0.66 and *t*_*Q*_ = 92.

The *first step* is to remove the impact of early mortality (infant, children, and young adults) to focus the analysis on the mortality of the most elderly. The rectangle is thus not drawn from birth, but from some pre-specified age, *t*_0 _(typically *t*_0_ = 50 years). The original survival curve S*(*t*) is thus replaced by a survival curve *S*(*t*) conditional on age ≥ *t*_0_, defined such that *S*(*t*_0_ ) = 1, and a high quantile *t*_*Q *_of the distribution of age at death conditional on age ≥ *t*_0 _is then considered, identified such that *S*(*t*_*Q*_) = 1 - *Q*. In order to get a more stable index, we do not use the maximal age at death (i.e., *Q* = 1), nor an extremely high quantile of the distribution (e.g., *Q* = .99). A good compromise is *Q* =.9. The lower-left and upper-right corner coordinates of the modified "moving rectangle" are now given by (*t*_0_, 1 - *Q*) and (*t*_*Q*_, 1), with a dimension (*t*_*Q*_ - *t*_0_)·*Q*. The proportion of the rectangle lying below the conditional survival curve (see Figure 1) defines the "rectangularity index" *R* such as:

The higher *R*, the more rectangular the survival curve. In particular, *R* = 1 if the survival curve is perfectly rectangular between *t*_0_ and *t*_*Q*_.

The *second step* is to define a "longevity index" as the quantile defined above, *t*_*Q*_. The higher *t*_*Q*_, the more shifted to the right the survival curve.

The *third step* is to recognize that life expectancy conditional on age ≥*t*_0_ and on age ≤ *t*_*Q*_, noted *CLE*_*t0,Q*_, is a simple function of the rectangularity index *R* and the longevity index *t*_*Q*_. Let *F*(*t*) = 1-*S(t*) and *f(t) = F'(t)* be the cumulative and density functions, respectively, of the conditional distribution of age at death. Integrating by parts, one obtains:

On the other hand, using the above definition of *R*, one has:

ending up with

In particular, for *t*_0_ = 0, *CLE*_*0,Q*_ = *R*·*t*_*Q *_is simply the product of both indexes.

From there, it is possible to decompose the differences between the *CLE*s of two survival curves as follows. Consider two curves with rectangularity indexes *R*(*A*) and *R*(*B*) and longevity indexes *t*_*Q*_(*A*) and *t*_*Q*_(*B*). The corresponding *CLE*s are:

and

The difference between the two *CLE*s can be expressed as:

Thus, any difference between two *CLE*s is a sum of two terms. The first term is the difference of *CLE*, which would be obtained if the longevity index would remain constant, set at the average of *t*_*Q*_(*A*) and *t*_*Q*_(*B*). The second term is the difference of *CLE*, which would be obtained if the rectangularity index would remain constant, set at the average of *R*(*A*) and *R*(*B*). In other words, the first term is interpreted as the part of the *CLE* difference attributable to a change of rectangularity, and the second term as the part attributable to a change of longevity.

**Figure 2:**
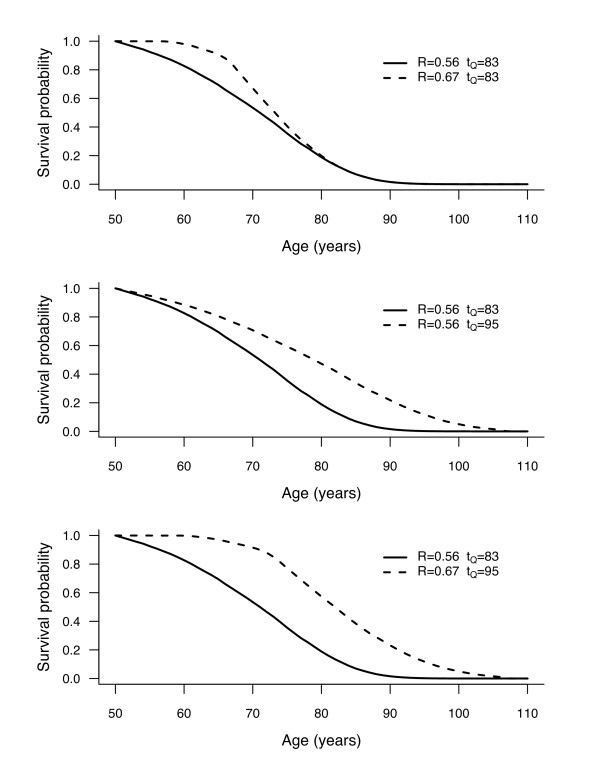
**Examples of conditional survival curves, where an increase of conditional life expectancy (for those persons reaching *t*_0 _ and dying before *t*_*Q*_, with *t*_0_= 50 and *Q* = 0.9) is due to rectangularization only (top panel), due to longevity increase only (middle panel), or due to both (bottom panel)**.

Hypothetical survival curves and patterns of change of *CLE* are shown in Figure 2, setting *t*_0_ = 50 years and *Q* = .9 in each panel. The solid line is a reference survival curve, established with *R* = .56 and *t*_*Q*_ = 83 years, thus with a corresponding *CLE*_50,0.9 _= 50 + .56(83 - 50) = 68.5 years. The dashed lines correspond to three different patterns of increase in *CLE* compared to the reference curve. In the top panel, rectangularity index *R* increased from .56 to .67, while *t*_*Q *_is stable at 83 years. In the middle panel, longevity index *t*_*Q *_increased from 83 to 95 years, while *R* is stable at .56. On the bottom panel, there is an increase of both the rectangularity index *R* (from .56 to .67) and the longevity index *t*_*Q *_(from 83 to 95 years).

Thus, in the top panel, the increase of *CLE* from 68.5 years to 50 + .67(83 - 50) = 72.1 years is due to an increase of rectangularity only. In the middle panel, the increase of *CLE* from 68.5 years to 50 + .56 (95 - 50) = 75.2 years is due to an increase of longevity only. Finally, in the bottom panel, the increase of *CLE* from 68.5 years to 50 + .67 (95 - 50) = 80.2 years is due to both an increase of rectangularity and an increase of longevity. There, the difference of 11.7 years can be decomposed as:

In other words, 4.3 years (37% of the overall gain in *CLE*) are attributable to a difference in rectangularity, while the remaining 7.4 years (63%) are attributable to a difference in longevity. In what follows, we shall refer to "life expectancy difference attributable to rectangularization" (*LEAR*), the part (in %) of difference in *CLE* due to a change in rectangularity (in this example, *LEAR* = 37% ).

## Results

The above approach has been applied to Swiss period life tables established by year of death for cohorts of men and women born in Switzerland between 1876 and 2006 [14]. The indicators above are implemented using *t*_0_ = 50 years and *Q* = .9.

**Figure 3:**
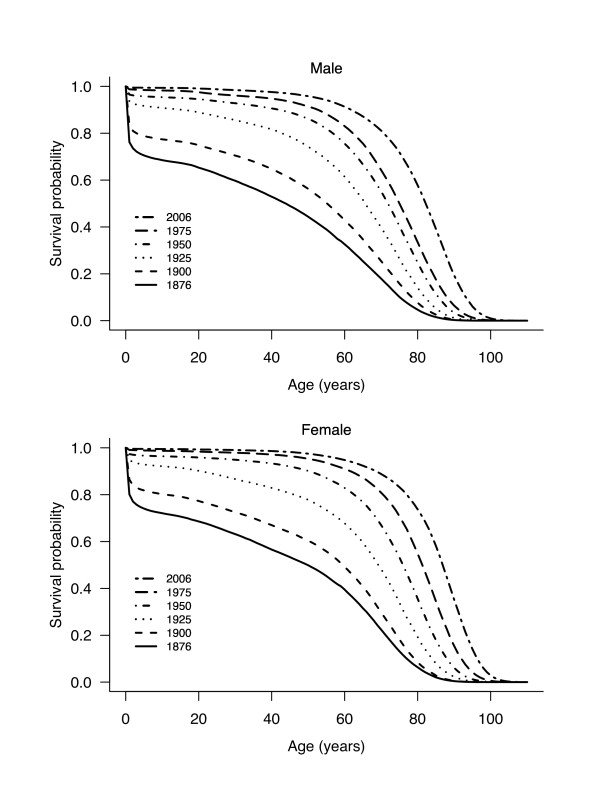
**Survival curves estimated from life tables by year of death (period) for cohorts of men (top panel) and women (bottom panel) born in Switzerland in 1876, 1900, 1925, 1950, 1975, and 2006**.

**Figure 4:**
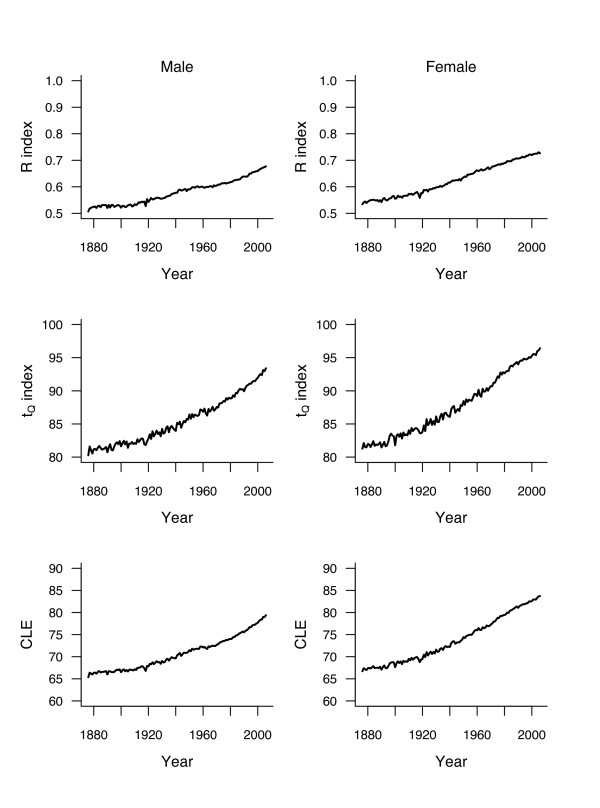
**Evolution of rectangularity index *R* (top panels) and longevity index *t*_*Q *_(middle panels), using *t*_0_ = 50 and *Q* = 0.9, estimated from life tables by year of death (period) for cohorts of men (left panels) and women (right panels) born in Switzerland between 1876 and 2006.** Bottom panels show the evolution of conditional life expectancy (for those persons reaching *t*_0_ and dying before *t*_*Q*_ ) during that period.

Figure 3 shows the survival curves for men and women for a sample of years. Rectangularity is visibly increasing over the century. This is reflected by an increasing *R* between 1876 and 2006, shifting from .51 to .68 for men and from .53 to .73 for women (upper panels of Figure 4). Figure 3 also shows a visible right shift of the curves, *t*_*Q *_increasing during this period from 80 to 93 years for men and from 81 to 96 years for women (middle panels of Figure 4). Overall, *CLE*_50,0.9 _increases by 14.1 years (from 65.3 to 79.4) for men and by 17.0 years (from 66.7 to 83.7) for women (bottom panels of Figure 4) between 1876 and 2006. Breaking up these differences shows that *LEAR* is similar in both sexes, slightly below 50% (namely, 45% for men and 44% for women). Thus, the increases in rectangularity and longevity contributed about equally to the increase in life expectancy.

**Figure 5:**
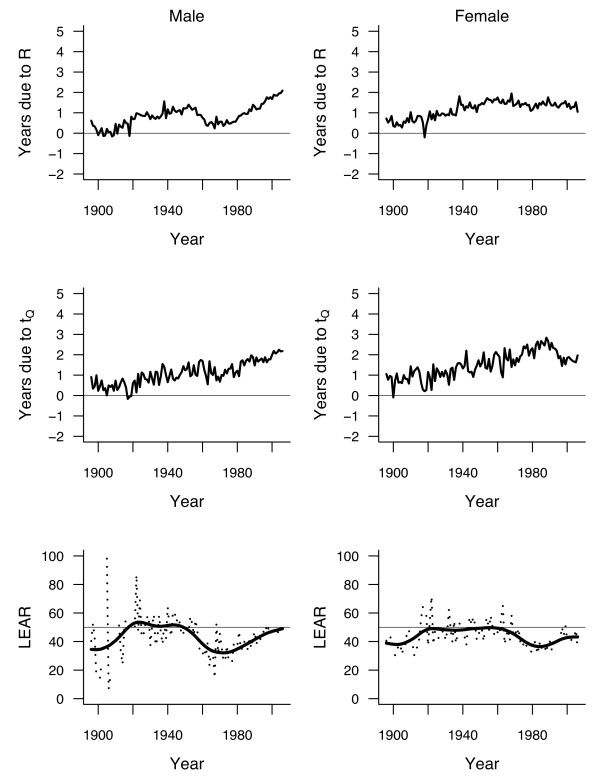
**Number of years gained (in conditional life expectancy compared to 20 years before) due to rectangularization (top panels) and to longevity increase (middle panels), based on indexes *R* and *t*_*Q*_with *t*_0_ = 50 and *Q* = 0.9, estimated from life tables by year of death (period) for cohorts of men (left panels) and women (right panels) born in Switzerland between 1876 and 2006**. The dotted lines in the bottom panels show the percentage of this gain attributable to rectangularization (*LEAR*). Smoothing splines have been added to show the trends (solid lines).

The above analysis of mortality is using only the first and last year of the observation period. In order to explore the variation of *LEAR* over the years, we compared the *CLE*_50,0.9 _of two curves 20 years apart, i.e., comparing 1896 to 1876, 1897 to 1877, etc., up to 2006 compared to 1986. Any other interval can be chosen, but a 20-year period is convenient because it corresponded approximately to one generation at the end of the 19th century. Eliciting shorter periods may lead to insignificant gains that are difficult to interpret. The secular evolution of *LEAR* from 1896 to 2006 (the ends of the first and of the last 20-year period considered, respectively) is shown in the bottom panels of Figure 5. Note that *LEAR* was not calculated for those few years with a decrease of either the rectangularity index or the longevity index. Smoothing splines [15] have been added to improve the readability of the graphs.

Overall, there were few changes over the years, with *LEAR* fluctuating around means slightly under 50% in both genders. Fluctuations were slightly more marked for men than for women, and more marked before 1920 (likely because of the stronger annual variation of mortality due to epidemics of communicable diseases). Overall, *LEAR* increased between 1876 and 1920, followed by a plateau and then by a decline since the 1940 s for men and since the 1960 s for women. *LEAR* was minimal (32%) for men in the 1970 s, and then increased slightly to below 50% at the turn of the century. For women, *LEAR* was minimal in the 1980 s at 36.5%, then increasing to 43% in 2006.

The same Figure 5 shows the secular trends in the number of years gained in 20 years attributable to rectangularization (top panels) and to longevity increase (middle panels). It is important to note that the rhythm of increase accelerated over the period, although there is a decline and a plateau for the figures for women.

Obviously, the same indicators can be used to compare the survival curves from two populations at a given moment. For example, applying these indicators to compare men and women, one finds that the difference in *CLE* was 4.3 years in 2006, with about half (2.2 years, *LEAR* at 51%) attributable to a difference in rectangularity.

## Discussion

This paper introduces the use of indicators characterizing the shape of a survival curve and measuring the impact of this shape on life expectancy. The first two indicators measure the rectangularity (*R*) and the longevity (*t*_*Q*_ ) of the survival curve at a given moment. The third indicator (*LEAR*) individualizes the impact of rectangularity and longevity on the differences in conditional life expectancy when comparing different moments or different populations. Unlike many others, our indicators are nonparametric and do not rely on any model, such as Gompertz [16], logistic [17], or Gompertz mortality change models [18].

The index of rectangularity is based on the "moving rectangle," an idea explored by Wilmoth and Horiuchi [5]. It depends on two parameters, *t*_0_ and *Q*, which will in turn affect the longevity index *t*_*Q*_ . The method can use any value of *t*_0_ and *Q*, although the values of the indicators are sensitive to this choice. When choosing *t*_0_ = 40 instead of *t*_0_ = 50, for example, for the Swiss mortality data from the previous section, the *LEAR* index comparing the years 1876 and 2006 is 48% for men and 46% for women instead of 45% and 44%. A similar comparison choosing *t*_0_ = 10 results in a *LEAR* index as high as 56% for men and 55% for women. The choice of the parameter values should relate to the question under study. If, for example, the interest lies in longevity and old age mortality, one should select a value for *t*_0_ high enough to remove premature mortality (largely due to accidents). In developed countries, a choice of *t*_0_ = 50 might be motivated by the fact that it is usually at that age that degenerative diseases (such as cardiovascular diseases or cancer) start to have some real impact. On the other hand, choosing *Q* = .9, i.e., removing the most elderly from the analysis, stabilizes the value of the longevity index (the index will not depend on a few persons, neither from the sample size if it is calculated using a sample of data instead of a life table). The resulting conditional life expectancy *CLE*_50,0.9_ is thus the mean age at death of those persons alive at 50 and dying before the 90^th ^percentile of age at death for this population. However, we do not exclude the possibility that other authors might wish to apply the method using other values of *t*_0_ and *Q*, depending on their interest. Obviously, one should keep the same values for these parameters when comparing mortality in two different countries, or when evaluating the trend of the *LEAR* index along the years. Note that the corresponding parameters for the life expectancy at birth would be *t*_0_ = 0 and *Q* = 1.

Other nonparametric indicators have been proposed in the literature to measure the various dimensions of lifespan and high longevity. In their review, Wilmoth and Horiuchi [5] analyzed 10 indicators to measure either the rectangularity of the survival curve or the variability of the distribution of age at death (coined as "moving rectangle", "fastest decline", "sharpest corner", or Keyfitz's H index), which were all highly correlated, such that these measures capture aspects of the shape of a survival curve that are strongly related. The authors concluded that one can choose one of them on the basis of convenience, and they favored the use of the interquartile range of age at death. Kannisto [6] further developed the idea, suggesting to consider the shortest age interval in which 50% of deaths take place as the best indicator of variability of age at death. Recognizing the need of having two indicators instead of one, Kannisto [7] finally proposed the modal age at death as a measure of longevity (which is in fact an alternative to our *t*_*Q*_ ), in combination with the standard deviation of the ages at death occurring above it as a measure of variability (an alternative to *R*). Canudas-Romo [19] also considered the modal age at death, although in combination with the standard deviation around (not above) the mode.

The indicators proposed by Kannisto [7] have been recently applied by Cheung et al [20] to analyze the evolution of life expectancy in Switzerland. With a decreasing standard deviation above modal age over the years, Cheung et al concluded to a significant compression of adult mortality, at least from 1920. However, because modal age at death was still increasing without respite, their analysis did not find any evidence that we are approaching longevity limits in terms of maximum life span. As seen in the Results section, we could find similar conclusions using our indicators.

One possible advantage of considering our indicators *R* and *t*_*Q *_instead of those of Kannisto is that they can be easily (almost visually) calculated given any survival curve. In contrast, modal age at death is known to be difficult to estimate, and its appropriate determination is crucial [7,20]. The most substantial single advantage of using our indicators, however, is that they can be naturally combined with each other, allowing the proposed decomposition of a change of life expectancy and hence to separate and to compare the effects of rectangularization and longevity increase to the secular increase of (conditional) life expectancy. This may be useful in the context of the debate initiated by Fries [3] as discussed in the Introduction.

## Conclusion

Analyzing the Swiss mortality data, our indicators suggest that the secular increase of life expectancy since the 1870 s is explained by the increase in both longevity and rectangularity, each responsible for about 50% of the increase. This corroborates Wilmoth's observation that both a rectangularization and an increase of longevity are simultaneously taking place [13]. It will be interesting to see whether the contribution of rectangularization to the secular increase of life expectancy will remain at around 50% or whether it will be increasing in the next few years. Also, a comparison among various industrialized and less industrialized countries with respect to this decomposition, as well as an analysis of the factors associated with the impact of rectangularization, or with the one of longevity increase on the decline of mortality, will be the subject of future research and should be of interest to many demographers.

## Competing interests

The authors declare that they have no competing interests.

## Authors' contributions

The approach proposed in this paper is the result of numerous discussions between FP, who submitted the problem, and VR, who developed an initial version of the method. Both authors participated in the writing and editing of the manuscript. Both authors read and approved the final manuscript.
